# Risks of synchronized low yields are underestimated in climate and crop model projections

**DOI:** 10.1038/s41467-023-38906-7

**Published:** 2023-07-04

**Authors:** Kai Kornhuber, Corey Lesk, Carl F. Schleussner, Jonas Jägermeyr, Peter Pfleiderer, Radley M. Horton

**Affiliations:** 1 Lamont-Doherty Earth Observatory, Columbia University, New York, USA; 2 Climate Analytics, Berlin, Germany; 3 German Council on Foreign Relations, Berlin, Germany; 4 Department of Geography and Neukom Institute, Dartmouth College, Hanover, USA; 5 Integrative Research Institute on Transformations of Human-Environment Systems (IRI THESys) and the Geography Department, Humboldt-Universität zu Berlin, Berlin, Germany; 6 Center for Climate Systems Research, Columbia University, Climate School, NY USA; 7 NASA GISS, Columbia University, New York, USA; 8 Potsdam Institute for Climate Impact Research, Potsdam, Germany

**Keywords:** Atmospheric dynamics, Climate-change impacts

## Abstract

Simultaneous harvest failures across major crop-producing regions are a threat to global food security. Concurrent weather extremes driven by a strongly meandering jet stream could trigger such events, but so far this has not been quantified. Specifically, the ability of state-of-the art crop and climate models to adequately reproduce such high impact events is a crucial component for estimating risks to global food security. Here we find an increased likelihood of concurrent low yields during summers featuring meandering jets in observations and models. While climate models accurately simulate atmospheric patterns, associated surface weather anomalies and negative effects on crop responses are mostly underestimated in bias-adjusted simulations. Given the identified model biases, future assessments of regional and concurrent crop losses from meandering jet states remain highly uncertain. Our results suggest that model-blind spots for such high-impact but deeply-uncertain hazards have to be anticipated and accounted for in meaningful climate risk assessments.

## Abstract (web summary)

Simultaneous harvest failures across crop-producing regions are major threats to global food security. A strongly meandering jet can trigger these, however, climate and crop models underestimate effects with consequences for climate risk assessments.

## Introduction

Extreme weather events like heatwaves, droughts and extreme precipitation can adversely impact crop production^1^ and food security^2,3^. Global warming is increasing the frequency and intensity^4–7^ of weather extremes and the likelihood of their simultaneous occurrence globally^8–10^. Extremes occurring in close temporal vicinity^11–13^ can lead to outsized societal impacts, often beyond the sum of each extreme occurring in isolation^14^. In particular, synchronized crop failures due to simultaneous weather extremes across multiple breadbasket regions pose a risk to global food security and food system supply chains^15,16^, with potential disproportional impacts for import-dependent regions^2,3^.

Risks of synchronized breadbasket failures have been assessed on a purely statistical basis^17,18^ and in relation to dominant modes of climate variability^16^ that act on annual and seasonal timescales. In the mid-latitudes, however, concurrent weather extremes are to a large degree driven by the jet stream, the fast flowing winds in the upper tropospheric mid-latitude circulation^19–21^. Specific summertime circulation regimes in the jet stream act as circumglobal teleconnections^22–24^, promoting simultaneous heat^9,25^ and rainfall^20,26^ extremes with adverse effects on agricultural production across the mid-latitudes^19^. In Northern Hemisphere (NH) summer, recurrent patterns have been identified as quasi-stationary Rossby waves with wavenumbers 5 and 7 (wave-5 and wave-7 from hereon), where the wavenumber refers to the number of ridges and troughs observed within the mid-latitudes, whenever their amplitude is high. Such high amplitude waves have been observed during major NH summer weather extremes recently^25,27,28^.

While earlier analyses have shown the importance of atmospheric wave patterns for local^29^ or coinciding^19,30^ extreme weather events, their impact on yield anomalies has to date only been quantified on a regional basis. The effect of such wave patterns on yield co-variability between pairs of regions remains unquantified in observations as well as in model experiments. Purely model based risk assessments require both crop models that show skill in reproducing observed extreme weather yield responses with sufficient accuracy and climate models that reproduce observed relationships between wave patterns and extreme weather. A recent analysis by Luo et al.^31^ shows that the surface imprint of wave patterns is largely underestimated in three climate models, while their phase position is reasonably well depicted. Recent CMIP6 models and bias-adjusted model experiments, however, have not been investigated. Thus, models on which recent risk assessments rely still lack a thorough review. Further, the performance of climate-crop models in simulating historical wave pattern-induced concurrent low yields across regions remains unassessed. In addition, potential future changes in wave patterns and associated surface anomalies and yield co-variability impacts have not been quantified so far.

Here, we map the observed concurrence patterns of low yields in major breadbasket regions associated with wave-5 and wave-7 patterns and probe the skill of models to reproduce the observed relationships. To do so, we use the most recent experiments from the Global Gridded Crop Model Intercomparison (GGCMI^32^) over five key crop regions that account for a large part of global maize and wheat production (maize: ~66%; wheat: ~70%^33^), and compare results based on historical model experiments to observed crop data^33^. To evaluate if current climate and crop models are suitable for credible multiple breadbasket risk assessments, we provide results from crop models driven by observations and crop models driven by bias-adjusted CMIP6 models (see data and methods section for details) over the historical time period (1960–2014). In doing so we aim at answering the following questions:

Estimating biases in climate models: How well are upper tropospheric jet patterns and associated surface weather anomalies reproduced in bias-adjusted CMIP6 simulations?

Regional impacts on crop yields in observation and models: Can a crop model driven by reanalysis data and bias-adjusted CMIP6 climate simulations reliably reproduce observed regional crop yield anomalies related to specific wave patterns?

Cross-regional impacts in observations and models: How do wave events modulate the concurrence of low yields in major crop producing regions, and how do results from the crop model driven by reanalysis and climate models compare to the observed signals of low yield concurrence?

The Results section is organized around answering these three categories of questions, primarily by analyzing observations and historical climate and crop simulations. However, our methodology also allows us to briefly offer projections towards the end of each of the three Results subsections, informed and in some cases tempered by important caveats identified in the comparison of historical simulations to observations.

## Results

### Circulation patterns and associated surface anomalies

**Fig. 1:**
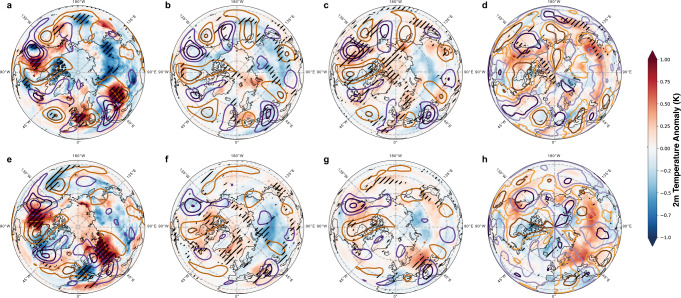
Circumglobal wave-7 and 5 patterns and associated 2 m air temperature anomalies in ERA-5 reanalysis data and bias-adjusted CMIP6 models. Meridional winds in m/s (contours; purple: southerly, orange: northerly winds, in (**a**–**c**, **e**–**g**) contours start at an absolute value of 3 m/s and increase/decrease by 3 respectively, in (**d**, **h**) contours start an absolute value of 0.5 and increase/decrease by steps of one) and near surface temperature anomalies filled contours during (**a**–**c**) wave-7 and (**e**–**g**) wave- 5 events relative to the respective climatology in the northern hemisphere summer (JJA) based on (**a**, **e**) ERA5 reanalysis (1960–2014), (**b**, **f**) historical (1960–2014) and (**c**, **g**) future (SSP5-8.5, 2045–2099) bias-adjusted output from CMIP6 simulations (four models). **d**, **h**) Difference in meridional winds and temperature response during wave events comparing historical and future patterns in four bias-adjusted CMIP6 models (for twelve non adjusted models see Fig. S6). Hatching shows statistical significance on a 95% confidence level (**a**, **d**, **e**, **h**) or 100% model agreement in sign (4 out of 4 models, **b**, **c**, **f**, **g**) While the phase positions and intensity of the wave patterns (line contour) are well represented in the models their surface imprint are considerably underestimated in historical simulations. Changes in the temperature response are identified over North America, Eurasia and East Asia (**d**, **h**).

While upper tropospheric wave patterns are well reproduced in the bias-adjusted CMIP6 multi-model mean, associated multi-model mean temperature (Fig. 1) and precipitation anomalies (Fig. S3, Table S1) are largely underestimated. To determine these composite maps wave events and anomaly fields are calculated for each model separately following the methodology of Kornhuber et al.^19^ (see methods: wave events) before averaging.

To quantify model agreement with reanalysis-data, we calculate the Pearson correlation *r* and coefficient of determination *R*^2^ of model-based composite fields (Fig.1 b, c, f, g) with those based on ERA-5 (Fig.1a, b) over the mid-latitudinal belt (38°−58°N). Note that *R*^*2*^ is calculated between the values of the two respective fields and not between values and a fit as is usually done. We find high values for meridional winds (${{cor}}_{v,{hist}}^{w7}=0.89,\,{R}^{2}=0.89$; ${{cor}}_{v,{ssp}585}^{w7}=0.92,\,{R}^{2}=0.92;{{cor}}_{v,{hist}}^{w5}$ = 0.82, ${R}^{2}=0.76$; ${{cor}}_{v,{ssp}585}^{w5}$ = 0.84, ${R}^{2}=0.8$), but low values for temperature anomalies in the multi-model mean (${{cor}}_{t2m,{hist}}^{w7}=0.35,\,{R}^{2}=0.14$; ${{cor}}_{t2m,{ssp}585}^{w7}=0.38,\,{R}^{2}=0.15;{{cor}}_{t2m,{hist}}^{w5}=$0.03, ${R}^{2}=0.02$; ${{cor}}_{t2m,{ssp}585}^{w5}=0.51,\,{R}^{2}=0.28$) and even lower values for precipitation anomalies (Table S1, Fig. S3). Luo et al.^31^ found similar results for three models partly based on CMIP5 experiments, suggesting that models might have not considerably improved in the newer CMIP6 experiments. Notably, bias-adjusted model outputs do not exhibit considerably improved spatial correlation values and anomaly fields compared to the original CMIP6 simulations (Table S2, Figs. S4, S5), possibly because bias adjustment optimizes fields for different subsamples rather than high amplitude wave events.

Circulation related changes in temperature during wave events comparing historical and future experiments for four bias-adjusted models are shown in Fig. 1, d, h. Future extreme warming simulations do not project an increase in mean wave amplitudes (Fig. S1), which have a tendency to be underestimated in historical experiments (Fig. 1). This is in line with no detectable trend in wave events over the historical time-period (Fig. S9).

However, ridges over certain regions do amplify in the future projections and contribute to larger positive temperature anomalies in the four bias-adjusted models (Fig. 1d, h, see Fig. S6 for anomalies based on all CMIP6 models). For wave-7, temperature anomalies increase significantly over Western North America (NA) and over East Asia (EAS), while for wave-5 significant increases are detected for Western NA and most of Eurasia. The correlations with observed temperature anomalies are higher for future compared to historical simulations in part due to increased temperature anomalies beyond mean warming alone.

**Fig. 2:**
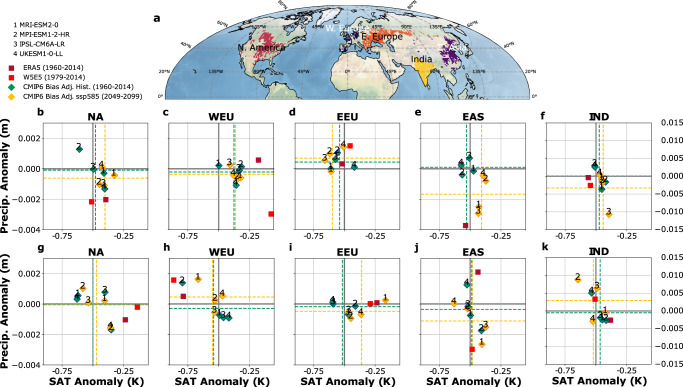
Mean response in precipitation and 2 m temperature anomalies over major crop-producing regions during wave events in reanalysis data and CMIP6 climate models. **a** Major crop producing regions in the Northern Hemisphere mid-latitudes defined by a threshold of 25% harvested area per grid-point. Weekly mean temperature and aggregated precipitation anomalies averaged over the regions outlined in (**a**) for (**b**–**f**) wave-7 and (**g**–**k**) for wave-5. We compare two different reanalysis datasets ERA-5 (dark red,1960–2014) and W5E5 (red, 1979–2014) with bias-adjusted output from four CMIP6 models under historical (green, 1960–2014) and future (2045–2099, SSP5-8.5, yellow) conditions, whereas their mean values are shown as dashed lines. Note the different *y*-axis range for (**k**) and (**f**) compared to the other panels. Temperature anomalies are dominantly underestimated in the bias-adjusted output in WEU (wave-7, wave-5), EEU and NA (wave-5). Precipitation anomalies are underestimated in NA, WEU (wave-7).

Concerning crop producing regions, the majority of the climate models analyzed here underestimate temperature and precipitation anomalies during wave events (Fig. 2). Underestimation of surface anomalies in models during wave events may translate into particular underestimated risk to crop productivity in key breadbasket regions. To assess this, we calculate the spatially averaged surface response in temperature and precipitation in important NH mid-latitude crop regions (Fig. 2a, see methods) during wave-7 and wave-5 events (Fig. 2b–k). We compare two different reanalysis datasets (ERA5, 1960–2014; W5E5, 1979–2014) to the bias-adjusted output from four CMIP6 models and their multi-model mean under historic and future simulations.

Strongest discrepancies between surface anomalies in reanalysis and models are found where the crop-producing regions spatially align with a wave-induced temperature anomaly, such as WEU for wave-7 and NA, WEU, and EEU for wave-5. For reanalysis, we find that NA shows above average temperatures during wave-5 events, and dry anomalies during wave-7 events. Strongest bivariate differences between wave patterns occur over western European (WEU) croplands which are wetter and colder during wave-5 events and hotter and drier during wave-7 events. Eastern Europe (EEU) exhibits warmer than average temperatures during wave-5 events and wetter than average conditions during wave-7 events. India (IND) and Eastern Asia (EAS) are drier than average during wave-7. Here, the reanalysis products covering the recent years (W5E5, both 1979-2014) exhibit wetter and drier conditions respectively, whereas ERA5 shows precipitation anomalies of opposite sign to the other reanalyses. The bias-adjusted model output does not exhibit a notable improvement relative to the other CMIP6 models over the historical period (Fig. 2, Fig. S7). Projected relative anomalies however do move towards the observed values in some regions, e.g. temperature anomalies for wave-5 in EEU and precipitation in IND for both waves (Fig. 2, Fig. S5), which aligns with the increased pattern correlation of future temperature anomaly fields identified earlier (Table S1).

### Observed and simulated regional yield losses

**Fig. 3:**
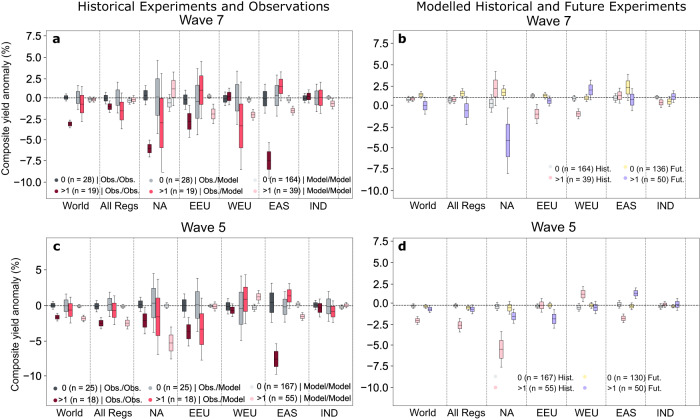
Combined Wheat and Maize yield anomalies during wave event years in observations and models. Composite yield anomalies based on wave events from ERA-5 reanalysis and annual reported national yield statistics from FAO (Obs/Obs), wave events from ERA-5 reanalysis and yield anomalies from a crop model (LPJmL) driven by reanalysis data (W5E5, Obs/Model) and crop model (LPJmL) driven by four bias-adjusted CMIP6 simulations (Model/Model) for (**a**) wave-7 and (**c**) wave-5 over the historical time period (1960–2014). Composites compare years in which two or more wave events are detected in JJA (red to purple bars) with the control case of years without such events (gray, light yellow bars). Bars and whiskers depict the distribution of 500 resampled replicate composite yield effects, where each replicated preserves the sample size of the underlying observations (wave events). Differences in detected wave events across datasets cause the difference in distribution variance. Differences in modelled crop impacts (both Obs/Model and Model/Model) are large compared to observations, but are smaller in some regions when driving the crop model with bias-adjusted reanalysis weather data (Obs/Model) instead of GCM simulations (Model/Model). (**b**, **d**) as in (**a**, **b**) but showing crop yield anomalies simulated by LPJmL based on historical (1960–2014) and future (SSP5-8.5, 2045–2099) simulations.

The occurrence of multiple wave events in summer negatively affects combined maize and wheat yield at the regional and global level in observational yield statistics (FAOSTAT^33^), but these impacts are not accurately reproduced by the crop-model experiments in most regions, especially when driven by bias-adjusted climate models (Fig. 3). We show this by comparing estimates of the waves’ composite impact on combined wheat and maize yield in observations (ERA5 x FAO-data: Obs/Obs), a crop model driven by a reanalysis product (W5E5 x LPJmL: Obs./Model) and the mean crop-model response from four climate models (bias-adjusted CMIP6 x LPJmL; Model/Model) under historical (Fig. 3a, c) and future conditions (Fig. 3b, d, see methods for further details).

We find that years with more than one wave event are associated with regional crop yield anomalies of up to −7% in EAS for, −6% to in NA and −3% in EEU for wave-7 with an average response across the selected regions of −2% to −3%. For wave-5 we observe values of −1% to −3% for respectively with highest regional anomalies observed in EEU (−3.7%) and NA (−2%) (Fig. 3a, c Obs/Obs). These results are in agreement with Kornhuber et al. 2020^19^, whose analysis relied on NCEP.NCAR reanalysis over a shorter period (1979–2018) and different crop data. Averaged across all regions, the response for wave-7 in CMIP6-driven experiments is essentially zero, which represents an underestimation of 3% globally relative to observations. While the yield anomalies averaged across all regions are in good agreement for wave-5, regional anomalies in models deviate from observed values: a 3% overestimation in NA compensates for the underestimation in all other regions. Historic crop simulations underestimate the response in EAS by 9% and by 3% in EEU (Fig. 3c).

In some regions, disagreements between purely observation and model-based assessments are substantially reduced when the crop model is driven with bias-adjusted ERA5 reanalysis data (W5E5, Fig. 1a Obs/Model). While disagreements with observed values remain over EAS and WEU, yield anomalies in NA, EEU and IND are found to be well within the error-margins of the observed impact. This provides evidence that surface anomaly biases in CMIP6 models are to a large degree responsible for ensuing crop model disagreement with observations in some regions. However, we note that this reduction in disagreement for the reanalysis-model hybrid is not consistent for all regions, suggesting regional variation in the accuracy of the crop model response to wave events.

Although linked climate and crop model simulations tend to underestimate observed crop impacts, a comparison of historical and projected future impacts might be still instructive given the large societal risks associated with even a small percentage change in yield. Future impacts on crop yield increase for wave-7 globally (Fig. 3b, d), and averaged over all regions, driven mainly by increased negative impacts in NA where yield is reduced by 6% compared to historical simulations, in agreement with the projected amplified heat response in that region in bias-adjusted model experiments (Fig. 1d, Fig. 2b). Meanwhile, wave-5 projected impacts dampen at aggregated geographic scales, primarily driven by much-reduced impact over NA that is only slightly offset by an increased impact over EEU. This increase over EEU is in agreement with the amplified temperature response observed in the multi-model mean for that region (Figs. 1h, 2i). We note that since the temperature and yield anomalies are normalized to respective time periods, these comparisons isolate the influence of wave patterns relative to contemporaneous climate, masking large mean warming and yield changes^32^ along with their own attendant uncertainties. Given the large biases in regional crop response to wave events, these results have to be considered with caution.

When quantifying the crop response to extreme weather events, their timing relative to the sowing and harvesting calendars is crucial^34,35^. For instance, the lack of wave event impact for wave events on WEU crop yield in observations might be related to dominance of winter wheat in this region, with a growing season extending far outside of JJA. Furthermore, we find that the timing of wave events within JJA differs between observations and CMIP6 (Fig. S8) presenting an additional potential explanation (beyond surface anomalies) for yield discrepancies between observations and CMIP6 models. While wave-5 events are evenly distributed over the summer season with a slight preference for the later weeks in ERA-5, these events skew towards the beginning of the season in the models, a tendency that is reinforced under a high emission scenario (Fig. S8). While wave-7 events predominantly occur in June and early July in ERA-5, CMIP6 models suggest a more evenly distributed occurrence of wave-7 events that reach into August.

### Observed and simulated concurrent yield losses

**Fig. 4:**
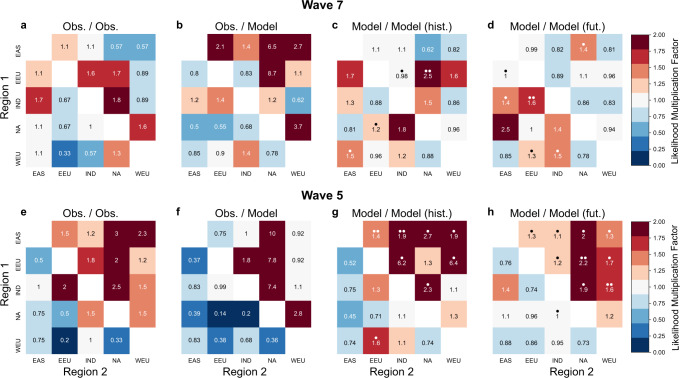
Likelihood multiplication factors (LMF) of concurrent yield losses in observations and models. LMF of concurrent negative yield anomalies of combined wheat and maize yield in two regions for wave-7 (**a**–**d**) and wave-5 events pear year (**e**–**h**) (upper right corner). LMF for concurrent positive yield anomalies are provided in the lower left corner of each heatmap. **a**, **e** Values based on ERA-5 and FAO data (1960–2014, Obs./Obs.). **b**, **f** Values based on wave events from ERA-5 and yield anomalies from LPJmL driven by W5E5 (Obs/Model.). LMF values for concurrent low and high yields differ significantly for (**a**, **b**, **e**, **f**) (Figs. S12–S15). Averaged LMF values based on LPJmL driven by four bias-adjusted CMIP6 models separately are shown for (**c**, **g**) historical experiments (1960–2014) and (**d**, **h**) future projections (2045–2099, SSP5-8.5). Model agreement is provided by dots where one dot indicates an agreement (above or below a LMF value of one) among three out of four models while two dots indicate an agreement among all four models.

The occurrence of one or more wave event in summer elevates the likelihood of poor harvests in pairs of two important crop producing regions in observations (Fig. 4a, e). To quantify the effect of wave events on concurrent poor yields we introduce the *likelihood multiplication factor* (LMF) in analogue to e.g. Zscheischler & Seneviratne^36^ (see Eq. (3) in Methods). The LMF is larger than one for a pair of two regions when wave events amplify the likelihood of concurrent low yields and lower than one when wave events have a lessening effect. Here we define a poor yield year as a year in which the combined wheat and maize yields are below the multiyear trend. In analogue we test the effect on concurrent yields above the long-term trends to test a potential beneficial effect on yield in two regions.

Results investigating concurrent negative anomalies are shown in upper right tiles of heatmaps in Fig. 4, while results on concurrent positive anomalies are presented in lower left tiles. In experiments based on observations (Obs./Obs., Fig. 4a, e) wave events increase the likelihood of concurrent negative yield anomalies in particular in pairings that include NA while they mostly decrease the likelihood of concurrent positive yield years overall. Both aspects are well represented in experiments based on LPJmL driven by reanalysis data (Fig. 4b, f), however values are higher for parings that include NA and EAS for wave-7. Further discrepancies between observation-based and modelled yields are found for pairings with WEU and EAS exhibiting overestimated LMF values for wave-7, while being mostly underestimated for wave-5. We find that LMF values for concurrent low yields and high yields differ statistically significant for both experiments (Obs./Obs., Obs./Model) and both waves throughout regions (Figs. S12–S15). Historical CMIP-6 models (Fig. 4c, g) exhibit a good agreement with observations, in particular for wave-5 where three out of four models agree in sign for most of the regions that have been identified as teleconnected. For wave-7 model agreement is strongest for the link between for NA x EEU, while the co-occurrence of positive yield anomalies is overestimated in some regions. This tendency increases for wave-7 in future projections, where concurrent positive yields dominate (Fig. 4d). The only pairings that see an increase in multi-model mean LMF for co-occurring negative yield anomalies are NA x EAS for wave-7 and NA x EEU and IND x WEU for wave-5. Notably, for future projections the four models exhibit poor agreement with respect to changes in low yield concurrence (compare Figs. S10, S11). While for wave-5 LMF values increase for MPI-ESM1-2-HR, values decrease in all regions besides NA for UKESM1-0-LL. Such disagreement in simulated concurrent yield loss across varying climate model inputs complicates drawing clear conclusions on on how LMF values will change in the future.

## Discussion

Concurrent crop failures in major crop-producing regions constitute a systemic risk as associated spikes in food prices can lead to conflict and undernutrition in countries that rely on imports^1,3^. Thus, understanding the likelihood of concurrent crop failures and the degree to which models are able to reproduce observed relationships is important for increasing the resilience of the global food system^15^ and mitigating climate risks.

While circulation patterns associated with high amplitude Rossby waves are accurately reproduced in climate models (Fig.1, Table S1), the magnitude of surface temperature and precipitation anomalies is largely underestimated, including in important crop producing regions (Figs. 1, 2). Similar results have been reported for other climate models mostly following the CMIP5 protocol^31^, however it is notable, that bias-adjusted output from CMIP6 experiments do not exhibit considerably improved spatial correlation and an accurate magnitude in surface anomaly fields (Fig. 1, Tables S1, S2), possibly because bias adjustment optimizes fields for different subsamples rather than high amplitude wave events.

Investigating future projections under a high emission scenario, we find no consistent global increase in wave amplitudes in models (Figs. S1, S2). This might be due to the fact that our wave diagnostic is applied to the mid-latitudes (37.5–57.5°N) and is therefore not sensitive to suggested^37^ changes in sinuosity in higher latitudes where the increased temperature gradient from increased land warming increases zonal winds and improves waveguidability^37,38^. Instead, lower wave amplitudes might be a consequence of the projected weakening of summertime stormtracks, associated with an increase in weather persistence^39,40^ in the mid-latitudes^39,41^, which might lower the magnitude of meridional winds on which the wave diagnostic is based on (see methods). We identify a regional amplification of troughs and ridges, in particular over the NA West coast and Eurasia (Fig. 1d, h, Fig. 2b, i, f) for wave-7 and wave-5 respectively. Amplified land-atmosphere feedbacks which are acting on top of regional circulation changes in a warmer climate^42–44^ are other potential factors for an increased regional temperature response. With the impacts of recent extreme heat events^45,46^ and associated wildfires^47,48^, such as the severe Pacific northwestern heatwave of 2021^45^ and the extraordinary Siberian heatwave of 2020^49^, these regions potentially emerge as high risk areas.

We find that simultaneous extremes linked to a meandering jet stream from amplified Rossby waves^19^ lead to regional yield losses (Fig. 3) and to concurrent low harvests across the mid-latitudes (Fig. 4). This increased likelihood of concurrent low yields in major breadbaskets, is mostly reproduced by historical model experiments, whether driven by reanalysis data or climate models in particular for wave-5 (Fig. 4). Regionally, however, we find that yield losses are mostly underestimated in crop models driven by climate model output (Fig. 3), while crop models driven by reanalysis data show more accurate responses.

The mostly adequate model representation of concurrent low yields combined with predominant underestimation of local impacts on yields, parallels the reasonable representation of modeled wave patterns and an underestimation of associated surface anomalies in bias-adjusted model output.

The biases in modelled crop yield response to wave event draw the reliability of future projections into question, but the societal importance of yield projections and the absence of a better approach argue for discussion under consideration of identified caveats. Increased local impacts on yields are identified in regions where future surface anomalies are projected to increase (e.g. NA for wave-7 and EAS for wave-5 (Fig. 2b, I, Fig. 3). However, the projected concurrence of poor yields, is found to be less conclusive (Fig. 4d, h), as models show divergent responses to future warming scenarios (Figs. S15, S16).

With positive trends observed in magnitude and frequency of extreme weather events, in particular for extreme heat, concurrent weather extremes causing interconnected and potentially disruptive impacts have become more common and will increase further if greenhouse gas emissions remain unmitigated^8,9,50^. Informed adaptation measures depend on models that simulate not just mean changes but also the changes in complex low probability but high-risk scenarios such as the concurrent and persistent extreme heat and rainfall extremes as observed e.g. in the extreme summers of 2018^25,51^ in Europe and Russia in 2010^28,52^, both with severe agricultural impacts^53,54^.

Assessing these complex risks depends upon an adequate representation of the location, magnitude, frequency and sub-seasonal distribution of extreme weather events, which may change under future emission scenarios. Our results highlight how the evolution of risks of multiple breadbasket failures under climate change are characterized by deep uncertainty in part due to the insufficient representation of the underlying climate impact drivers in models^55^. Other major observational and modelling uncertainties regarding climate change risks to global crop production include the magnitude of the CO2 fertilization effect and general changes in the hydrologic cycle. Our results point to an additional modelling uncertainty with import specifically for inter-regional crop yield covariability, which have unique consequences for the global food system. Future work should examine potential interactions among these key uncertainties, particularly the potential modulation of jet-related hazards by mean hydrologic and thermodynamic change.

While climate models have been excellent in projecting the mean response to continued anthropogenic greenhouse gas emissions^56^, our analysis suggests that they might provide a conservative estimate of how concurrent extreme weather events driven by specific circulation regimes might evolve in future and how they might affect regional crop yield and covariability across regions. Further we highlight that the underestimation of surface extremes identified in CMIP5 models^31^ and impacts on yields from their bias-adjusted output still persist in most recent climate and crop simulations. Physically constrained machine learning methods designed to maintain patterns and coherence across variables might offer an effective tool for an improved bias adjustment for more accurate impact assessments^57^.

Our study points towards potential high-impact blind spots in current climate risk assessments, highlighting the urgent need for more empirical and process-based research to support model improvements in the climate and agriculture domains, supplemented by expert elicitation, qualitative storylines^58^, and decision-centric approaches^59^. Evidence for high-risk blind spots such as an underestimation of synchronized harvest failures as identified here, manifests the urgency of rapid emission reductions, lest climate extremes and their complex interactions might increasingly become unmanageable.

## Methods

### Data

ERA5^60^ reanalysis data for years (1960–2014) data using the recently published back-extension was downloaded from the Copernicus Data Store (https://cds.climate.copernicus.eu/#!/home). Daily mean fields of 2 m temperature, precipitation and meridional winds are based on hourly data at timesteps 0:00, 6:00, 12:00 and 18:00. Downloaded at a 0.25 × 0.25 resolution, the data was re-gridded to 1 × 1 resolution. Temperature and precipitation data was detrended by subtracting linear trends on a grid-point basis over the investigated period and a subsequent subtraction of the day-in-year climatological value over the same period in order to remove the seasonal cycle. For temperature and meridional winds weekly anomalies are calculated by averaging daily mean data centered around the day for which a high amplitude is detected. For precipitation, weekly anomalies are calculated by a 7-day aggregation of daily anomalies centered around the day for which an event is detected. Meridional winds are analyzed a 250 mb level.

W5E5^61^ is the primary input data for the ISIMIP3-project, is based on bias-adjusted ERA5 data for the period 1979-2014 and GSWP3 homogenized to W5E5 for years 1960-1978 and was acquired from the internal repository of the Potsdam Institute for Climate Impact research. For a more detailed description about the bias adjustment process see^62^ and https://www.isimip.org/gettingstarted/input-data-bias-correction/details/80/. Wave events for Fig. 2 and Figs. S4, S5 are based on ERA5 wind-fields. As years prior to 1979 are not based on ERA5 we limit the analysis to years 1979–2014.

CMIP6^63^ daily 2-meter temperature, precipitation and meridional wind fields were retrieved from the *google data store* and re-gridded to 1 × 1° resolution for years 1960–2014 (historical runs) and 2045-2099 (SSP5-8.5). Temperature and precipitation fields were detrended following the approach described above. Precipitation fields from CMIP6 were converted from precipitation flux (kg m^−2^ s^−1^) to meter per unit cell per hour by multiplying each value with a factor of 3.6. Models included in this analysis were chosen based on the availability of meridional wind fields on daily time-scale for both time-periods investigated. The following twelve models were included: BCC-CSM2-MR, CESM2-WACCM, CNRM-CM6-1, CNRM-ESM21, CanESM5, EC-Earth3-Veg, GFDL-CM4, IPSL-CM6A-LR, MIROC6, MPI-ESM1-2-HR, MRI-ESM2-0, UKESM1-0-LL.

### Bias-adjusted models and yield-data

We identify maize and wheat responses to amplified wave patterns based on a 3-tier protocol: (i) FAO^33^ crop yield statistics compared with reanalysis weather data, (ii) crop model-based yield estimates driven by reanalysis weather data, (iii) crop model-based yield estimates based on 4 downscaled and bias-adjusted CMIP6 climate models. Our analyses leverage crop model simulations facilitated by AgMIP’s Global Gridded Crop Model Intercomparison (GGCMI)^32^. Here we use global LPJmL simulations at 0.5° for maize and wheat driven by W5E5^61^ reanalysis data (1960-2014) and climate model simulations from 4 CMIP6 GCMs (2045-2099), bias-adjusted and downscaled by ISIMIP^64^ (IPSL-CM6A-LR, MPI-ESM1-2-HR, MRI-ESM2-0, UKESM1-0-LL) to estimate the effect of high amplitude waves on crop yield anomalies. For more details on the crop modelling protocol, see Jägermeyr et al.^32^.

Crop producing regions and regional aggregation: Crop-producing regions in the northern hemisphere mid-latitudes are defined similar to prior studies: North America (United States, Canada), Western Europe (France, Switzerland, Spain, Portugal, United Kingdom, Belgium, Netherlands, Germany), Eastern Europe (Greece, Bulgaria, Moldova, North Macedonia, Ukraine, Romania, Serbia, Albania, Russia), India and Eastern Asia (China, Mongolia). Grid-points relevant for crop production for the analysis shown in Fig. 2 are defined by a grid-point based threshold of 25 % harvested area fraction based on 2005 fields from Ray et al.^65^. We further include an ‘all regions’ grouping including all wave event-affected countries, as well as global total yield levels as a ‘world region’ which refers to the global yield level in a given year. We aggregate crop yield data to the regional scale by summing national FAO production and divide it by total harvested area in each year, and summing gridded modeled crop production data, which are based on static harvested area over time. Because the crop model cannot simulate annual variations in harvested area, we strictly compare simulated yield (i.e., productivity) with observational yield levels from FAO. We then simply detrend modeled and observed crop yields using singular spectrum analysis (SSA), a non-parametric method that avoids assumptions about the functional form of the climate and yield trends^66^. This isolates interannual yield variability from longer-term trends, and thus quantifies yield variations relative to ~10-year moving windows. Thus, the composites reflect yield variations relative to contemporaneous yields, enabling us to composite wave event impacts relative larger long-term mean changes in yield.

### Wave analysis and wave event definition

We analyze wave patterns following the approach from Kornhuber et al.^19^, decomposing weekly averages of the weighted mean of midlatitude (37.5°N–57.5°N), meridional wind fields (250 mb) with a fast Fourier decomposition in June-August (JJA). The 92 days in the JJA yield 13 weeks. The weekly averaging assures some degree of anti-aliasing, and serves as a low-pass filter. High amplitude wave events are detected when the weekly amplitude exceeds a value of 1.5 standard deviation above the mean relative to the respective wave’s JJA climatology. As events are based on a threshold that varies with the respective climatology, we find similar event numbers for each dataset. For ERA5 that is 54 wave-7 events and 62 wave-5 for JJA 1960–2014 (see Fig. S9 for number of events per year for each wave).

### Composite analysis

For each tier in the protocol described above, we use a compositing method similar to Kornhuber et al.^19^ to estimate the regional composite yield anomalies for both years with >1 wave event, and years with no wave events (the ‘control’). The method isolates the signal of specific events in noisy time series by averaging anomalies across the sample of either >1 or 0 wave event years within each tier of the protocol (i.e., observed or modeled future/historical wave events). Prior to averaging regional yield values for the sample of >1 or 0 event years, the values are divided by the average yield values for the three years preceding and following the event, generating standardized composites (in terms of % of regional production). To estimate uncertainty in this point estimate of the standardized composite yield anomaly, we re-estimate 500 replicates of the composites using ~90% of the full sample. This bootstrap approach yields a distribution of resampled composites, which we depict as box-and-whisker plots. Values mentioned in the results reflect median composite estimates across the 500 replicates. Further details on the compositing method can be found in Kornhuber^19^.

### Likelihood multiplication factor and definition of poor yield years

The LMF for a specific wave and a pair of regions is determined by first calculating the number of years that feature concurrent below average yields *y1* and *y2* in those specific regions conditioned on the occurrence of more than one wave event of the specific wavenumber ${N}_{\left(y1 < 0,y2 < 0\vee w > 1\right)}$ divided by the total number of years that feature more than one wave event per year ${N}_{w > 1}$:

1 $${p}_{w > 1}={N}_{\left(y1 < 0\cap y2 < 0\vee w > 1\right)}/{N}_{w > 1}$$

To determine the LMF we calculate the ratio of ${p}_{w > 1}$ and

2 $${p}_{0}={N}_{\left(y1 < 0\cap y2 < 0\vee w=0\right)}/{N}_{w=0,}$$

the ‘hit rate’ of concurrent low yields conditioned on the occurrence of no wave event:

3 $${{{{{{\rm{LMF}}}}}}=p}_{w > 1}/{p}_{0}.$$

Thus, LMF provides a factor by which the occurrence of wave events increases the occurrence of concurrent low yields in two regions, relative to years in which no events occur. In Fig. 4 we provide results on concurrent high yields in analogue to this approach. Poor or good yields years are defined as those years in which a region exhibits combined wheat and maize yields below or above the multiyear trend respectively. This moderate threshold ensures a more reliable statistic through a higher sample size. The following example is based on ERA-5 and FAO data and regions WEU and NA and wave 7: we find ${N}_{\left(y1 < 0,y2 < 0\vee w > 1\right)}=$6 years where yields are below the long-term trend in both regions that also feature more than one wave event. Throughout the dataset ${N}_{w > 1}=15$ years are identified in which more than one wave event occurs, thus: ${p}_{w > 1}=\frac{6}{15}$. Of those, only one event shows concurrent positive yield anomalies. In contrast, we find ${N}_{w=0}=20$ zero event years of which ${N}_{\left(y1 < 0\cap y2 < 0\vee w=0\right)}=5$ feature concurrent low yields. Thus ${p}_{0}$=5/20. For wave-7 and NA x WEU we find:

$${LMF}=\frac{6}{15}\,{{\cdot }}\,\frac{20}{5}=1.6.$$

During years with more than one wave-7 event the probability of concurrent low yields is increased by a factor of 1.6 or 60% compared to years with no wave-7 event (see Fig. 4a).

## Supplementary information

Supplementary Information

## Data Availability

Reanalysis and CMIP6 datasets used in this study are publicly available at https://cds.climate.copernicus.eu/#!/home and https://esgf-node.llnl.gov/search/cmip6/ respectively, the GGCMI datasets are available at https://agmip.org/aggrid-ggcmi/.

## Code availability

All scripts relevant for the presented analysis will be made available at GitHub: https://github.com/KaiKornhuber/BreadBaskets_code.git.
