# Supplementary material for: Risks of synchronized low yields are underestimated in climate and crop model projections
Source: Nat Commun. 2023 Jul 4;14:3528. doi: 10.1038/s41467-023-38906-7 (PMC10319847; doi:10.1038/s41467-023-38906-7)
Supplement: Supplementary file 1 — Supplementary Information [file 41467_2023_38906_MOESM1_ESM.pdf]

Risks of synchronized low yields are underestimated in climate and crop model projections

Kai Kornhuber[1,2,3*], Corey Lesk[1,4], Carl F. Schleussner[2,5], Jonas Jägermeyr[6,7,8], Peter Pfleiderer[2,5], Radley M. Horton[1,2]

[1]Lamont-Doherty Earth Observatory, Columbia University, New York, USA
[2]Climate Analytics, Berlin, Germany
[3]German Council on Foreign Relations, Berlin, Germany
[4]Department of Geography and Neukom Institute, Dartmouth College, Hanover, USA
[5]Humboldt University Berlin, Berlin, Germany
[6]Center for Climate Systems Research, Columbia, New York, USA
[7]NASA GISS, Columbia University, New York, USA
[8]Potsdam Institute for Climate Impact Research, Potsdam, Germany
*Corresponding author: kk3397@columbia.edu

Supporting Information

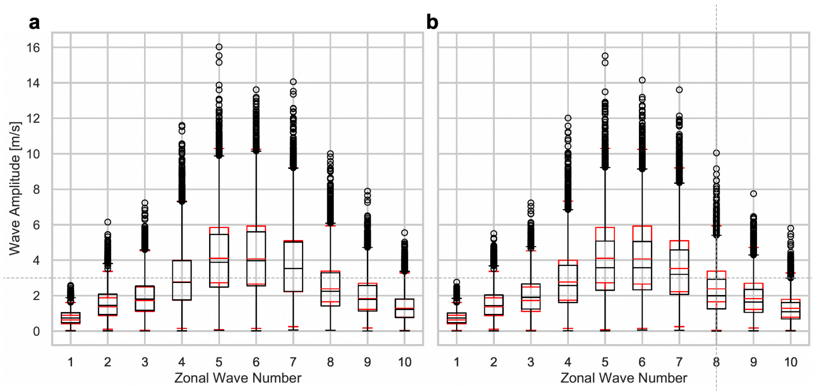

Figure S1 Wave amplitudes in Northern hemisphere Summer in CMIP6 models under **a** historic (1960-2014) and **b** future (ssp585, 2045-2099) scenarios compared to spectra based on ERA5 (1960 – 2014) shown as boxplots, where boxes illustrate the interquartile range, the vertical line the median, whiskers the 75th (25th) percentile plus (minus) 1.5 time the interquartile range. Outliers are shown as circles.

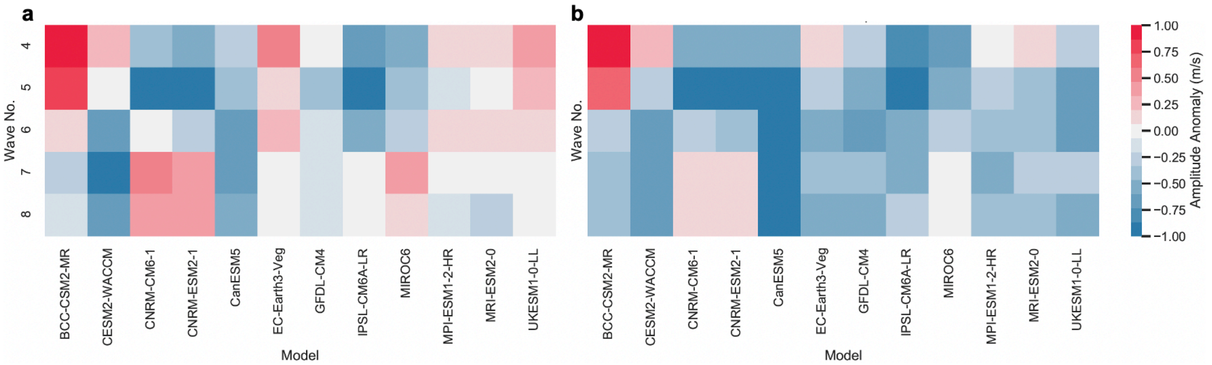

Figure S2 Wave amplitudes in Northern hemisphere Summer in CMIP6 models under **a** historic (1960-2014) and **b** future (ssp585, 2045-2099) scenarios compared to spectra based on ERA5 (1979 – 2019).

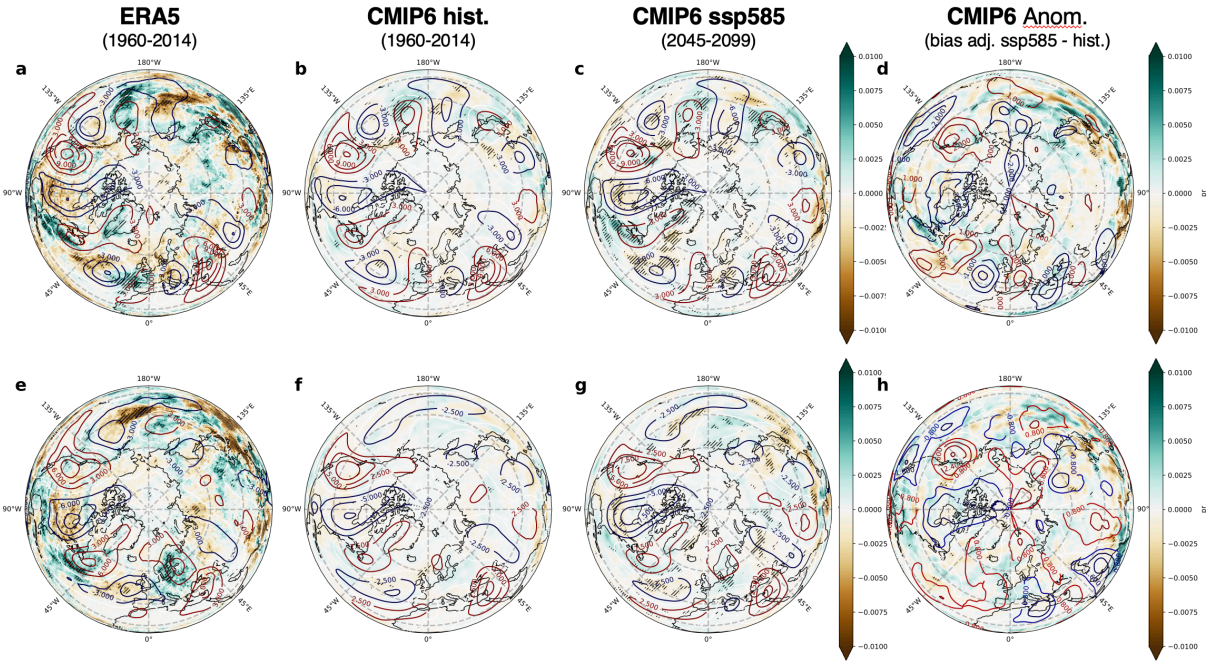

Figure S3 Circumglobal wave 5 and 7 patterns and associated precipitation anomalies in ERA-5 reanalysis data and CMIP6 models. Similar to figure 1 but for rainfall: Meridional winds (contour) and precipitation anomalies during **a-c** wave-7 and **e-g** wave- 5 events relative to the respective climatology in the northern hemisphere summer (JJA) based on **a, e** ERA5 reanalysis (1960-2014), **b, f** historical (1960-2014) and **c, g** future (ssp585, 2045-2099) CMIP6 simulations (twelve models). **d, h** Difference in meridional winds and precipitation response during wave events comparing historical and future patterns in four bias-corrected CMIP6 models. Hatching shows statistical significance on a 95% confidence level (a,d,e,h) or 75% model agreement in sign (9 out of 12 models, b,c,f,g) While the phase positions and intensity of the wave patterns are well represented in the models, their surface imprint are considerably underestimated in historical simulations. Regional changes in response are identified by the end of the century **d,h**.

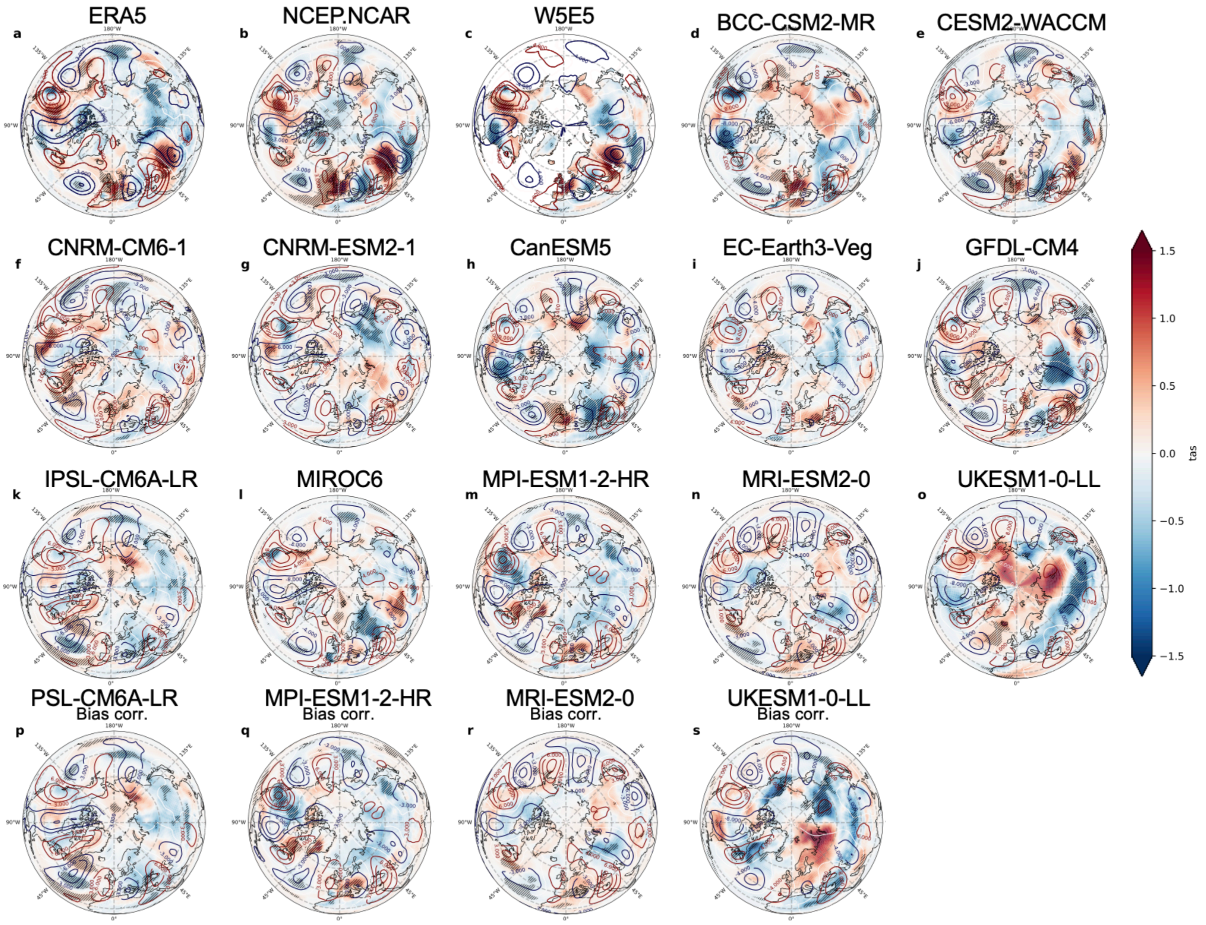

Figure S4 Circumglobal wave-7 patterns in v-winds (contour) and associated surface temperature anomalies in **a-c** reanalysis data (a-c), **d-o** CMIP6 models **p-s** and bias adjusted CMIP6 models.

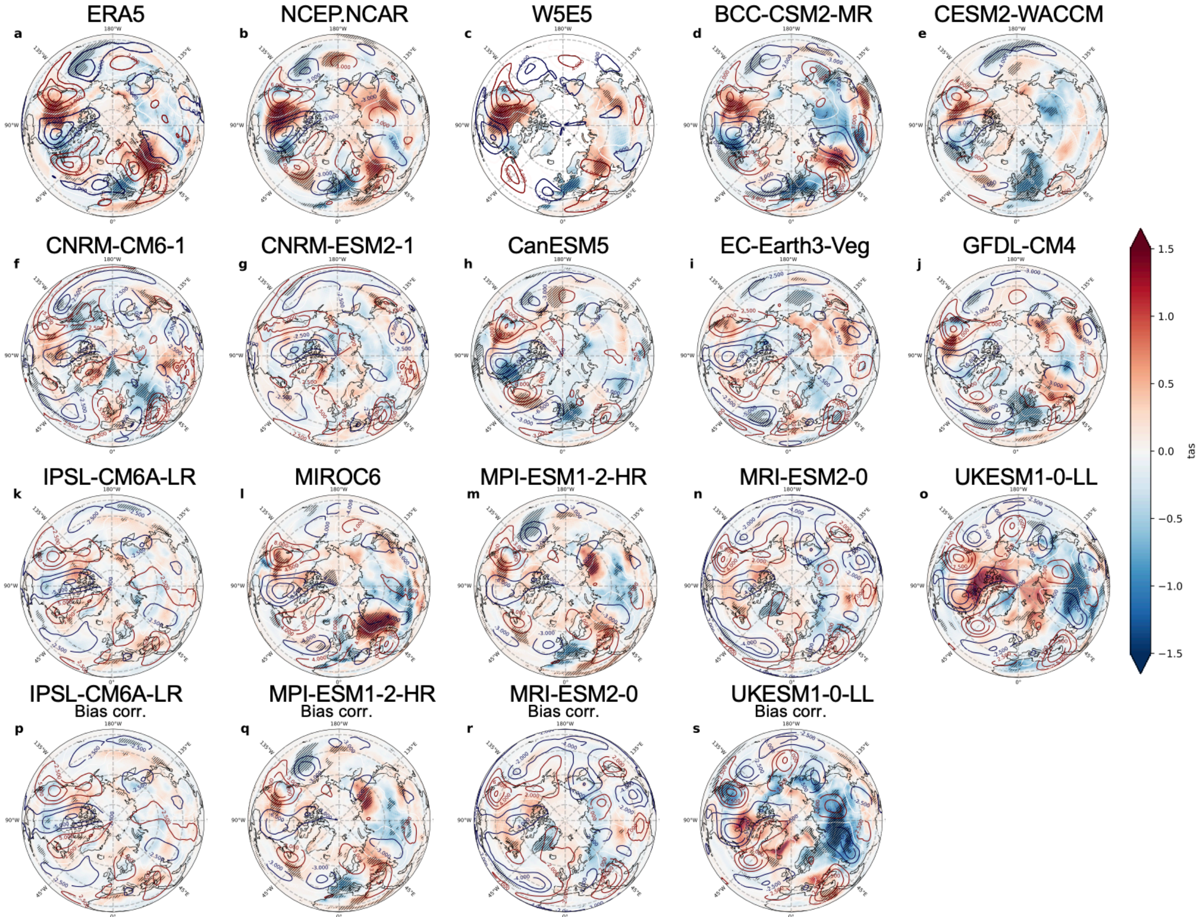

Figure S5 Circumglobal wave-5 pattern in v-winds (contour) and associated surface temperature anomalies in **a-c** reanalysis data (a-c), **d-o** CMIP6 models **p-s** and bias adjusted CMIP6 models.

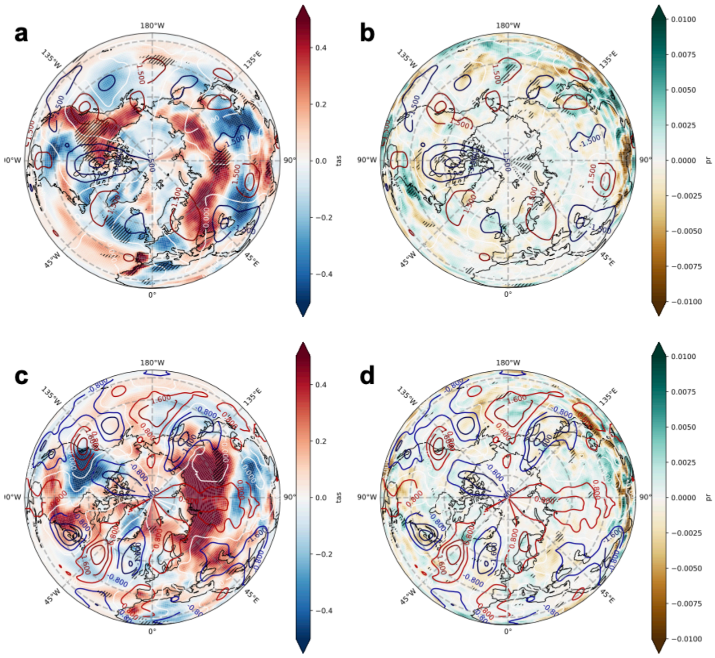

Figure S6 Changes in meridional winds (250mb), **a,c** surface temperature and **b,d** precipitation during **a,b** wave-7 and **c,d** wave-5 events between historical (1960-2014) and future (ssp585, 2045-2099) CMIP6 experiments.

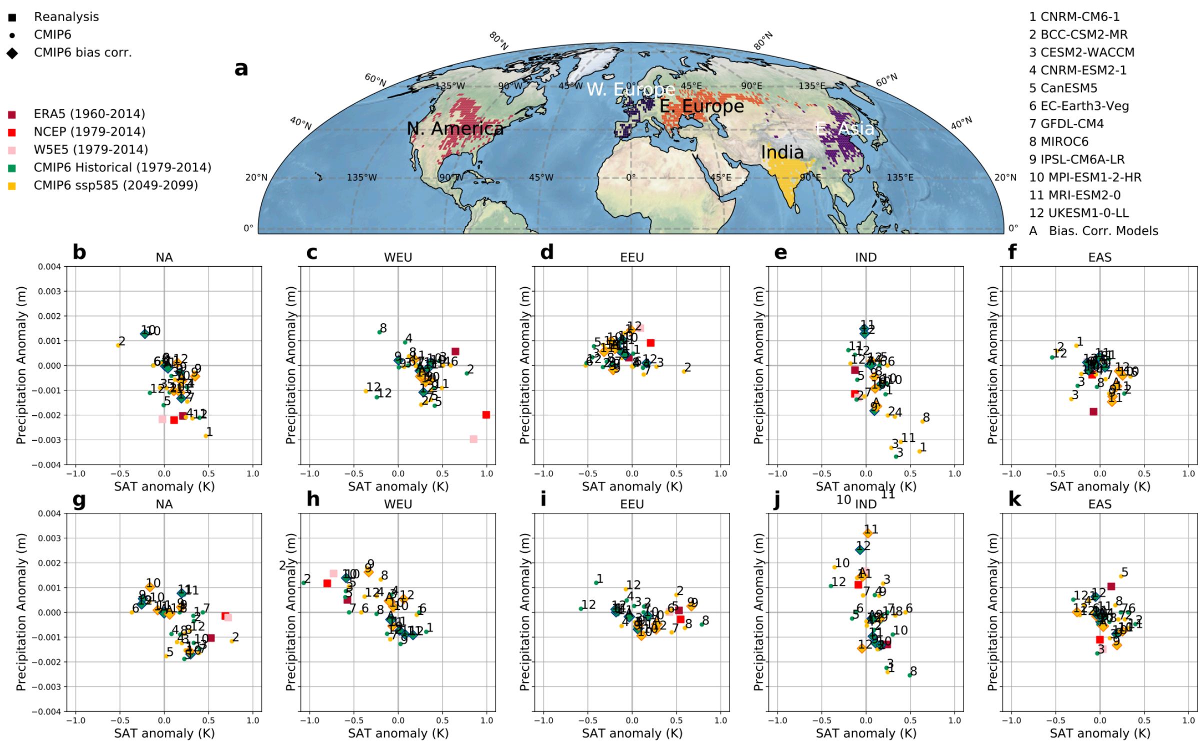

Figure S7. As Figure 2 but including results from future (ssp585) model experiments (yellow). **a** Regions of major crop production in the northern hemisphere mid-latitudes. Weekly mean temperature and aggregated precipitation anomalies averaged over the regions outlined in **a** for **b-f** wave-7 and **g-k** for wave-5. We compare three different reanalysis datasets ERA-5 (dark red,1960-2014), NCEP.NCAR (red, 1979-2014) and bias corrected W5E5 (pink, 1979-2014) with twelve CMIP6 models under historical (green, 1960-2014) and future (2045-2099, ssp585, yellow) conditions. The four bias corrected models are plotted as diamonds. Precipitation values are multiplied by 0.5 for regions IND and EAS so that y-axes can be shared. Temperature anomalies are underestimated in CMIP6 and CMIP6 bias corrected models in WEU (wave-7, wave-5), EEU and NA (wave-5). Precipitation anomalies are underestimated in NA, WEU (wave-7).

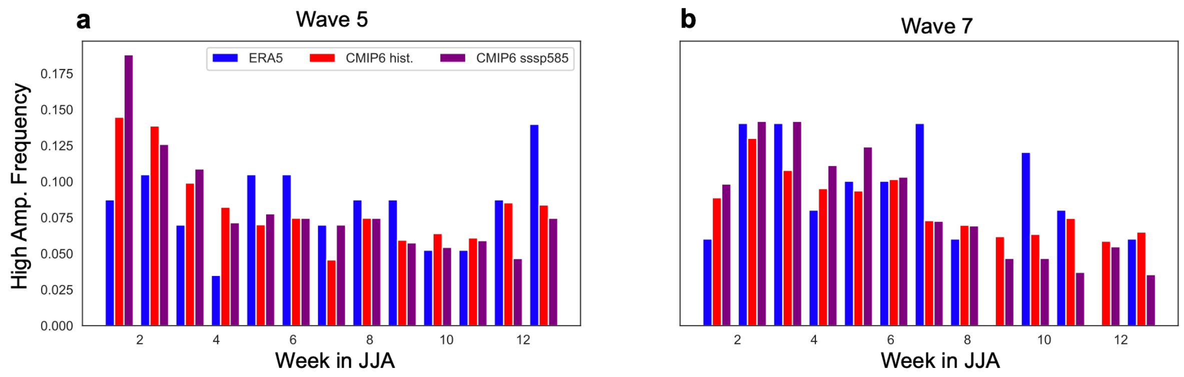

Figure S8 Seasonality of **a** wave 5 and **b** wave 7 in ERA5 and the four bias adjusted CMIP6 models employed for the crop experiments under historical and future conditions. Some differences between seasonal distribution are observed.

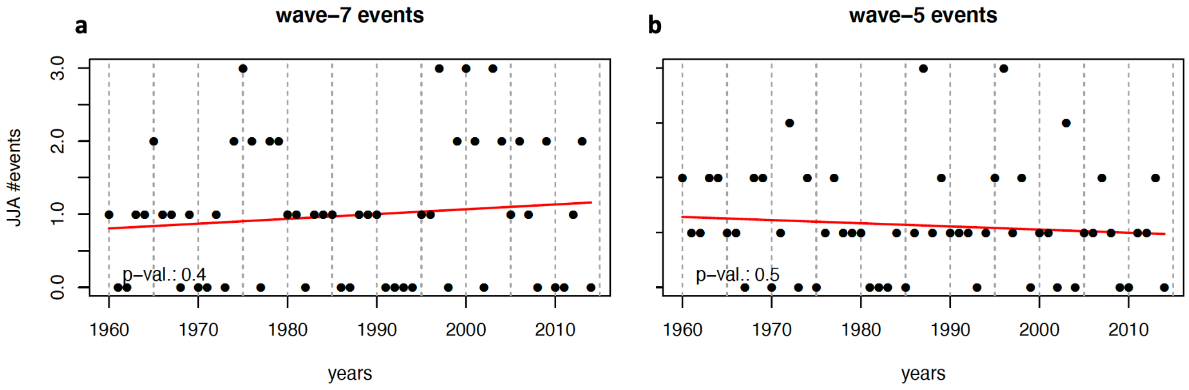

Figure S9 Events per year for of **a** wave-7 and **b** wave-5 in ERA5 within the 13 weeks in JJA throughout years 1960-2014. A linear regression (red solid line) shows respective linear trends that are found to be statistically insignificant considering a 95% confidence interval (see p-values in lower left corner, respectively).

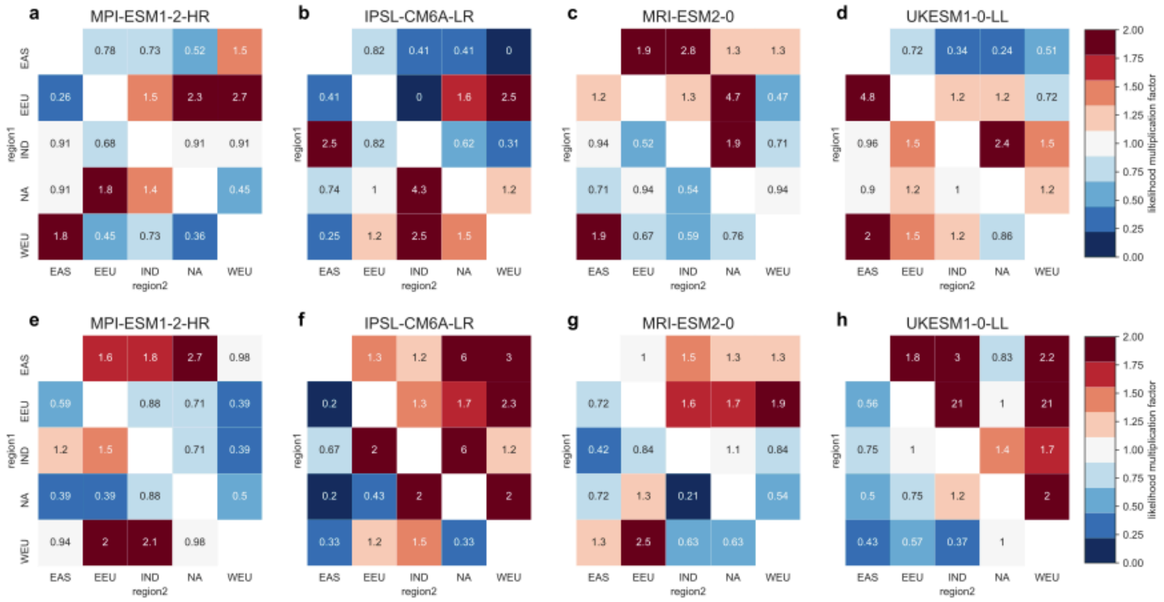

Figure S10 Likelihood multiplication factor of combined wheat and maize yield in LPJmL driven by historical simulations of four bias corrected CMIP6 models. LMF values of concurrent negative yield anomalies of combined wheat and maize yield in two regions for wave-7 (**a-d**) and wave-5 event pear year (**e-h**) (upper right corner). LMF for concurrent positive yield anomalies are provided in the lower left corner of each heatmap.

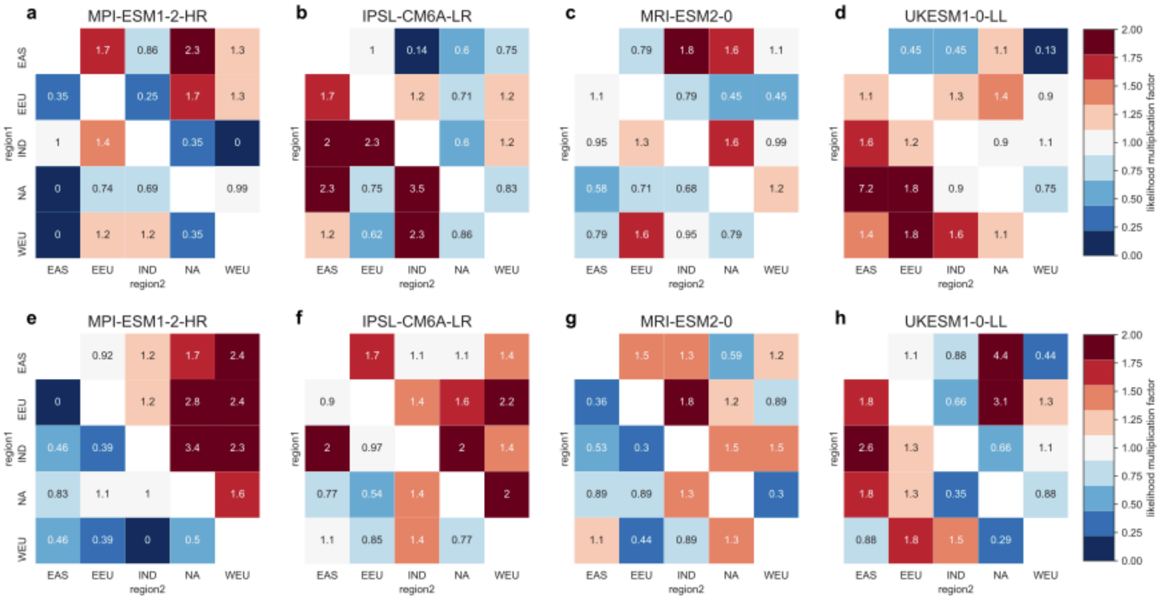

Figure S11 Likelihood multiplication factor of combined wheat and maize yield in LPJmL driven by a future high emission scenario of four bias corrected CMIP6 models. LMF values of concurrent negative yield anomalies of combined wheat and maize yield in two regions for wave-7 (**a-d**) and wave-5 event pear year (**e-h**) (upper right corner). LMF for concurrent positive yield anomalies are provided in the lower left corner of each heatmap.

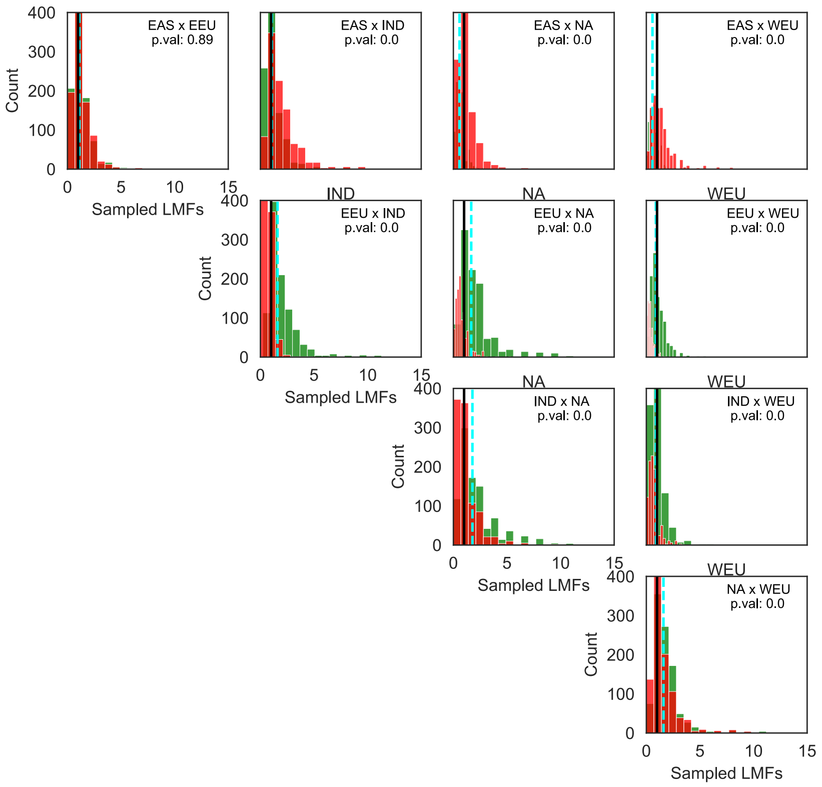

Fig. S12 Distributions of randomly sampled (N=1000) Likelihood multiplication factors (LMF) for the case of concurrent above average yields (red) and concurrent below average years (green) in two regions for wave 7 and ERA-5 (compare Fig. 4 a). Black lines indicate an LMF of 1, whereas the dashed cyan line gives the values shown in

the upper right corner of Fig. 4. P-values are based on a Kolmogorov-Smirnov test applied to the two samples. A value of 0.0 implies a p-value of below 1e-3.

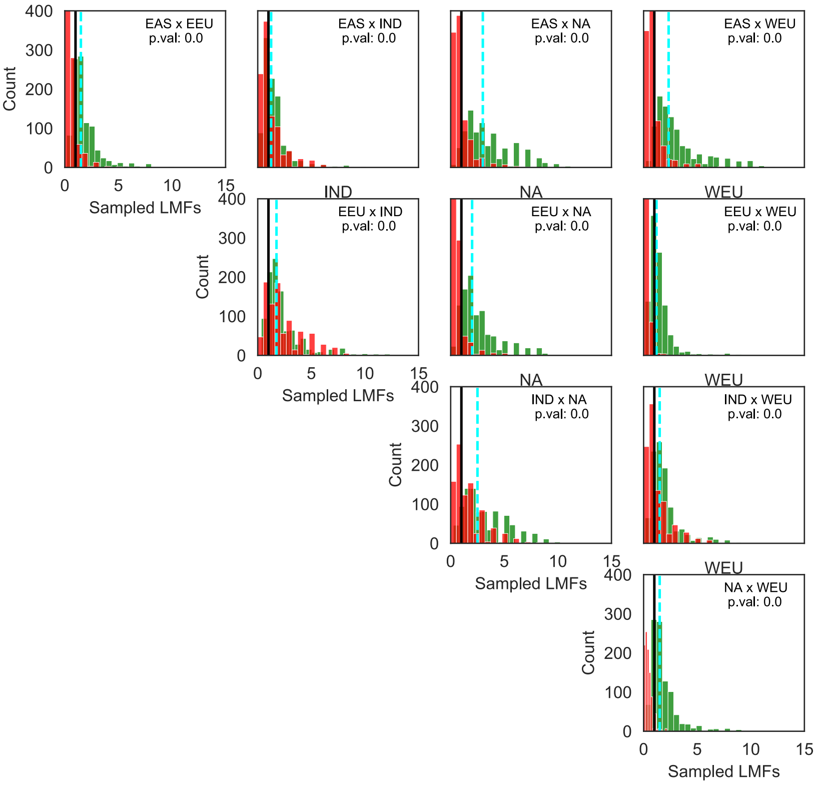

Fig. S13 As in Fig. S12 but for Wave 5 (compare Fig. 4e).

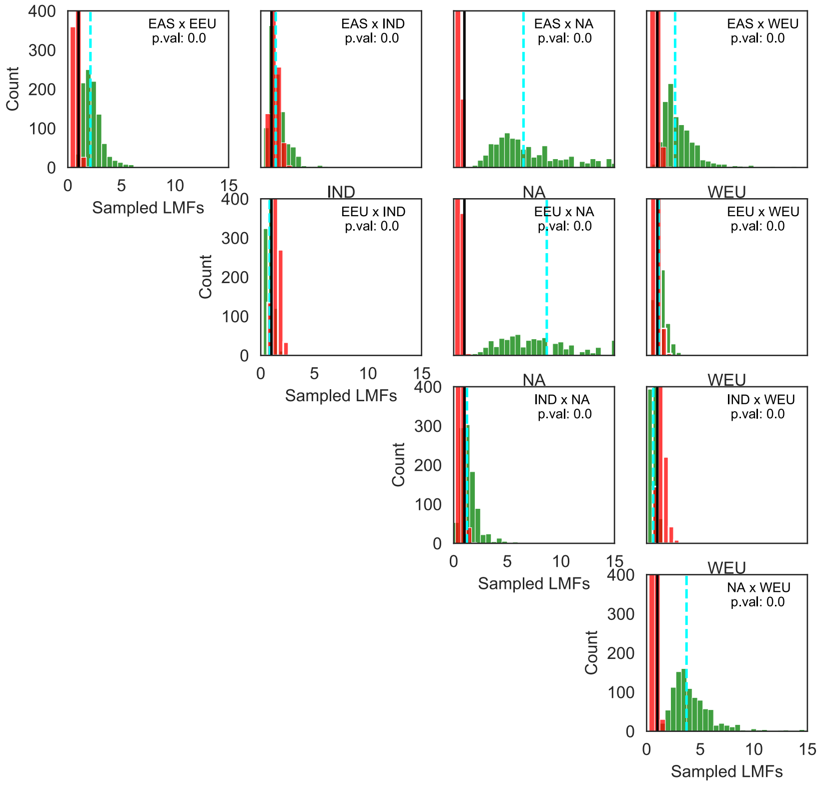

Fig. S14 As in Fig. S12 but for Wave 7 and for W5E5 (compare Fig. 4 b).

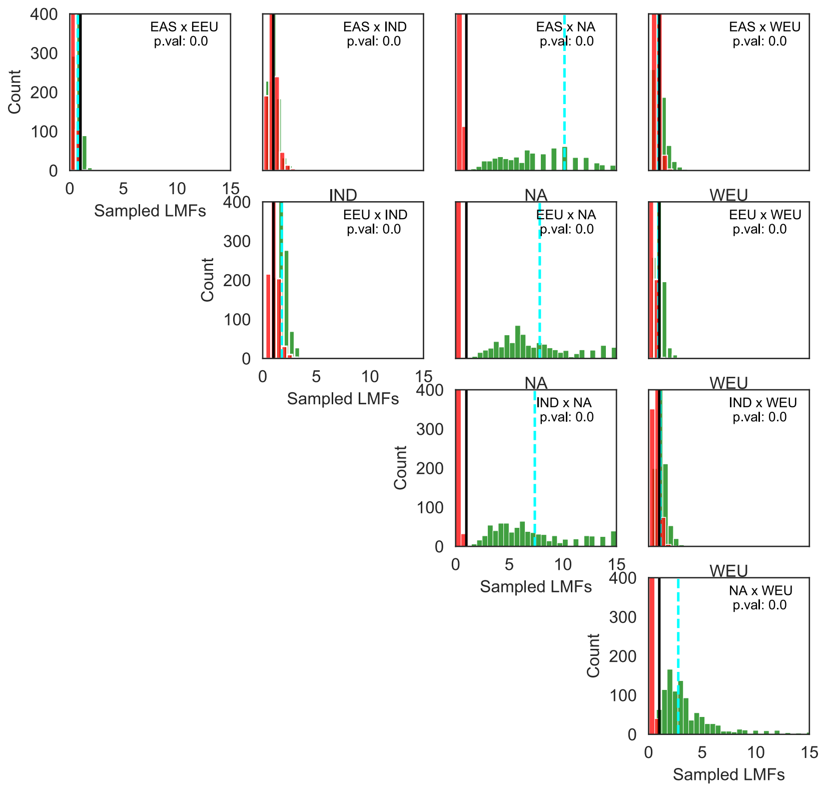

Figure S15 As in Fig. S12 but for Wave 5 and for W5E5 (compare Fig. 4 f).

Tables

		Wave -7							Wave-5					
Model	Experiment	v-wind corr.	R^2	SAT Corr.	R^2	Precip. Corr.	R^2		v-wind corr.	R^2	SAT Corr.	R^2	Precip. Corr.	R^2
Model Mean	historical	0.89	0.89	0.35	0.14	0.27	0.16		0.82	0.76	0.03	0.02	0.00	0.00
	ssp585	0.92	0.92	0.38	0.25	0.27	0.20		0.84	0.80	0.51	0.28	0.19	0.13
IPSL-CM6A-LR	historical	0.75	0.75	0.58	0.39	0.17	0.16		0.62	0.58	-0.19	-0.14	-0.12	-0.12
	ssp585	0.80	0.80	0.16	0.14	0.22	0.20		0.78	0.77	0.46	0.35	0.14	0.13
MPI-ESM1-2-HR	historical	0.89	0.89	0.27	0.17	0.17	0.16		0.90	0.89	0.59	0.57	0.31	0.31
	ssp585	0.91	0.91	0.50	0.44	0.17	0.17		0.83	0.81	0.27	0.23	0.16	0.14
MRI-ESM2-0	historical	0.84	0.84	-0.21	-0.17	0.13	0.12		0.47	0.41	-0.26	-0.20	-0.12	-0.11
	ssp585	0.92	0.92	0.45	0.38	0.18	0.17		0.42	0.40	-0.18	-0.13	0.05	0.05
UKESM1-0-LL	historical	0.88	0.86	0.07	-0.02	0.05	0.04		0.77	0.77	-0.18	-0.26	-0.18	-0.17
	ssp585	0.83	0.82	-0.13	-0.13	0.06	0.05		0.83	0.83	0.36	0.30	0.01	0.01

Table S1 Pearson correlation values of comparing meridional wind, temperature and precipitation anomaly fields during high amplitude wave-5 and wave-7 events based on ERA-5 and the for bias corrected CMIP6 models under historical conditions

		Wave 7							Wave 5					
Model	Experiment	v-wind corr.	R^2	SAT Corr.	R^2	Precip. Corr.	R^2		v-wind corr.	R^2	SAT Corr.	R^2	Precip. Corr.	R^2
Model Mean	historical	0.94	0.94	0.58	0.35	0.59	0.37		0.87	0.83	0.72	0.44	0.29	0.13
	ssp585	0.91	0.91	0.67	0.41	0.52	0.35		0.88	0.85	0.77	0.52	0.47	0.29
BCC-CSM2-MR	historical	0.92	0.92	0.35	0.31	0.45	0.43		0.83	0.83	0.75	0.72	0.34	0.32
	ssp585	0.89	0.88	0.42	0.36	0.34	0.33		0.92	0.88	0.65	0.59	0.39	0.37
CESM2-WACCM	historical	0.91	0.91	0.61	0.57	0.38	0.36		0.91	0.91	0.58	0.55	0.31	0.30
	ssp585	0.85	0.84	0.00	-0.06	0.46	0.46		0.88	0.87	0.14	0.11	0.31	0.31
CNRM-CM6-1	historical	0.75	0.75	0.26	0.23	0.33	0.31		0.23	0.23	-0.12	-0.11	-0.04	-0.04
	ssp585	0.69	0.69	0.33	0.29	0.32	0.31		-0.02	-0.02	-0.24	-0.19	0.03	0.02
CNRM-ESM2-1	historical	0.67	0.67	0.36	0.30	0.15	0.13		0.23	0.21	-0.03	-0.02	0.07	0.06
	ssp585	0.69	0.68	-0.01	-0.01	0.03	0.03		0.28	0.27	0.07	0.06	0.03	0.01
CanESM5	historical	0.91	0.91	0.53	0.45	0.44	0.44		0.93	0.93	0.52	0.49	0.32	0.31
	ssp585	0.82	0.79	0.39	0.34	0.24	0.22		0.89	0.89	0.49	0.48	0.40	0.39
EC-Earth3-Veg	historical	0.90	0.89	0.23	0.16	0.33	0.28		0.69	0.67	-0.18	-0.17	-0.23	-0.21
	ssp585	0.89	0.89	0.37	0.37	0.27	0.26		0.74	0.73	0.51	0.48	0.17	0.16
GFDL-CM4	historical	0.90	0.90	0.22	0.20	0.54	0.52		0.87	0.86	0.60	0.58	0.27	0.26
	ssp585	0.93	0.92	0.72	0.72	0.59	0.58		0.90	0.90	0.70	0.69	0.34	0.33
IPSL-CM6A-LR	historical	0.75	0.75	0.55	0.37	0.19	0.17		0.62	0.58	-0.20	-0.15	-0.11	-0.10
	ssp585	0.80	0.80	0.15	0.13	0.18	0.17		0.78	0.77	0.44	0.33	0.12	0.12
MIROC6	historical	0.91	0.88	0.45	0.40	0.32	0.30		0.86	0.83	0.61	0.58	0.07	0.06
	ssp585	0.87	0.86	0.48	0.41	0.15	0.15		0.76	0.73	0.52	0.48	0.05	0.04
MPI-ESM1-2-HR	historical	0.89	0.89	0.26	0.16	0.14	0.12		0.90	0.89	0.59	0.58	0.28	0.27
	ssp585	0.91	0.91	0.48	0.43	0.15	0.14		0.83	0.81	0.26	0.22	0.13	0.11
MRI-ESM2-0	historical	0.84	0.84	-0.25	-0.21	0.11	0.10		0.47	0.41	-0.25	-0.20	-0.13	-0.11
	ssp585	0.92	0.92	0.45	0.38	0.19	0.17		0.42	0.40	-0.18	-0.13	0.05	0.05
UKESM1-0-LL	historical	0.88	0.86	0.08	-0.06	0.03	0.03		0.77	0.77	0.07	0.03	-0.18	-0.16
	ssp585	0.83	0.82	0.01	0.01	-0.05	-0.05		0.83	0.83	0.39	0.35	0.08	0.07

Table S2. Pearson correlation coefficients of composite v-wind (250mb), surface temperature anomaly and precipitation anomaly fields during wave 5 and wave 7 events detected in ERA-5 (1960-2014) and historical (1960-2014) and future (2045-2099, ssp585) CMIP6 model simulations. Modelled meridional wind patterns match the reanalysis patterns well, while the surface response in temperature and precipitation is reproduce to a lesser degree.
